# Multi‐Omics Evidence Linking Gualou Xiebai Banxia Decoction Intervention to Atherosclerosis Mitigation and Gut Microbiota–Bile Acid Signatures in ApoE ^−/−^ Mice

**DOI:** 10.1002/fsn3.71543

**Published:** 2026-02-16

**Authors:** Rutao Bian, Li Zhang, Jun Zhu, Xuegong Xu

**Affiliations:** ^1^ Zhengzhou Hospital of Traditional Chinese Medicine Zhengzhou China; ^2^ Zhengzhou Hospital of TCM Affiliated to Henan University of Chinese Medicine Zhengzhou Hospital of Traditional Chinese Medicine, Henan University of Chinese Medicine Zhengzhou China

**Keywords:** atherosclerosis, bile acids, cholesterol catabolism, Gualou Xiebai Banxia Decoction, gut microbiota

## Abstract

Atherosclerosis presents a persistent health challenge, with limited therapies addressing residual cardiovascular risk. Gualou Xiebai Banxia Decoction (GXBD), a classical Chinese herbal formula traditionally used for chest obstruction syndromes, was evaluated as a dietary‐style intervention in ApoE^−/−^ mice fed a high‐fat diet for 14 weeks. Using a multi‐omics strategy that combined UHPLC‐QE‐MS/MS chemical profiling, network pharmacology, 16S rRNA sequencing, targeted bile acid metabolomics, and biological validation, we assessed vascular and metabolic outcomes alongside gut ecology. Chemical profiling identified 348 constituents, including bioactive flavonoids and saponins. In vivo, GXBD intervention significantly improved lipid profiles by reducing serum TC, TG, and LDL‐C, and by raising HDL (*p* < 0.05). It markedly reduced aortic plaque burden and alleviated hepatic steatosis (*p* < 0.05). Mechanistically, GXBD reshaped the gut microbiota, characterized by the enrichment of beneficial *Bacteroides* and *Alloprevotella* and the depletion of pro‐inflammatory *Blautia* and *Bilophila*. This microbial shift coincided with significantly higher levels of protective secondary bile acids, such as 11‐LCA and 23‐DCA, and fewer cytotoxic chenodeoxycholic acid–derived metabolites (*p* < 0.05). Correlation and constrained ordination analyses linked these microbial‐bile acid signatures to the concordant modulation of vascular hub targets, including the downregulation of *MMP9* and *CASP3* and upregulation of *PPARG* and *SIRT1*. These findings suggest that GXBD mitigates atherosclerosis in this murine model through a coordinated remodeling of the gut microbiota–bile acid–host axis, supporting its potential as a microbiome‐informed, multi‐component adjunct for cardiometabolic health.

## Graphical abstract

Gualou Xiebai Banxia Decoction (GXBD) promotes atherosclerotic plaque stabilization via the “gut microbiota—bile acid—host” axis. GXBD treatment remodels the gut microbiota, promoting the production of secondary bile acids (11‐LCA and 23‐DCA). These metabolites enter the systemic circulation and modulate vascular gene expression—specifically upregulating *PPARG* and *SIRT1* while downregulating *MMP9* and *CASP3*—thereby enhancing fibrous cap thickness and reducing plaque vulnerability.

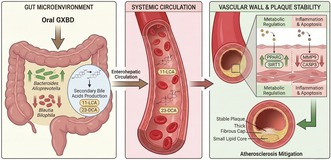

## 1 Introduction

Atherosclerosis (AS) remains the principal cause of cardiovascular disease and global morbidity, posing a significant public health burden (Schumski et al. 2021). While HMG‐CoA reductase inhibitors have dramatically reduced incidence by targeting hyperlipidemia, a large proportion of patients still face considerable residual cardiovascular risk (Szántó et al. 2021). This risk is predominantly driven by non‐traditional factors, including chronic inflammation, endothelial dysfunction, and increasingly, dysbiosis of the gut microbiota (Kondapalli et al. 2025; Shi et al. 2025). Therefore, novel therapeutic strategies that modulate these non‐lipid pathways are urgently needed to reduce the global burden of AS further (Fusco et al. 2025).

Traditional Chinese Medicine (TCM) offers a rich repository of multi‐component therapeutic formulas with established efficacy against complex diseases such as AS. Gualou Xiebai Banxia Decoction (GXBD) is a classic formula initially documented in the *Synopsis of Prescriptions of the Golden Chamber* (*Jingui Yaolue*). Clinically, GXBD is the core treatment for *Xiong Bi* (Chest Obstruction), a syndrome characterized by chest pain and oppression, which aligns with modern coronary heart disease and AS. TCM theory posits that *Xiong Bi* is linked to the accumulation of “Phlegm Turbidity” (*Tan Zhuo*), essentially reflecting systemic metabolic and circulatory dysfunction. Preliminary modern studies have confirmed GXBD's protective effects against hyperlipidemia (Zhang et al. 2024) and myocardial ischemia (Lin et al. 2025). However, the precise multi‐target mechanism by which this complex herbal mixture counteracts the systemic pathology of AS remains largely unexplored.

The bidirectional interaction between the gut microbiota and the host has emerged as a critical regulator of AS pathogenesis (Schoeler and Caesar 2019). Gut microbes perform essential metabolic functions, notably converting primary bile acids (BAs) into secondary BAs, such as lithocholic acid (LCA) and deoxycholic acid (DCA) derivatives (Collins et al. 2023). Beyond aiding lipid digestion, BAs act as signaling molecules that influence metabolic homeostasis and vascular health. Dysbiosis‐driven BA alterations increase intestinal permeability, elevate circulating endotoxins, and exacerbate vascular inflammation, forming a feed‐forward loop that accelerates plaque progression (Vancamelbeke and Vermeire 2017; Zhang et al. 2023). Therefore, investigating whether the holistic pharmacological action of a multi‐component TCM formula, such as GXBD, involves the unique modulation of this axis is of significant scientific merit.

In this study, we employed an integrated multi‐omics workflow—combining chemical profiling, network pharmacology, 16S rRNA sequencing, and targeted bile acid metabolomics—to systematically define the anti‐atherosclerotic mechanism of GXBD (Li et al. 2025). Our goal was to establish a clear link between the chemical components of GXBD and its ability to alleviate AS in ApoE^−/−^ mice, hypothesizing that the gut microbiota–secondary bile acid–host metabolic axis serves as a crucial therapeutic pathway. These findings will not only provide robust scientific evidence for the modernization of GXBD but also propose a microbiome‐targeted adjunctive strategy for managing cardiovascular residual risk.

## 2 Materials and Methods

### 2.1 Preparation of GXBD Extract

GXBD was prepared according to the classical prescription ratio (Table 1) using high‐quality, authenticated *Trichosanthis Fructus* (Gualou), *Allii Macrostemonis Bulbus* (Xiebai), and *Pinelliae Rhizoma* (Banxia). The herbs were subjected to three successive decoctions in purified water (at volumes of 20, 12, and 12 times) at 100°C for 60 min each. The combined filtrates were concentrated under reduced pressure, lyophilized, and stored at −20°C. The consistency and reproducibility of the extract were validated by determining the extract yield and standardized by UHPLC‐QE‐MS/MS profiling.

**TABLE 1:** Herbal components of GXBD.

Chinese name	Plant name	Part used	Proportion (g)
Gualou	*Trichosanthis* *Fructus*	Fruits of *Trichosanthes kirilowii* Maxim	12
Xiebai	*Allium macrostemon Bulbus*	Bulb of *Allium macrostemon* Bge	9
Banxia	Pinelliae Rhizoma	Tuber of *Pinellia ternata (Thunb)* Breit	9

### 2.2 Chemical Profiling by UHPLC‐QE‐MS/MS

A 100 mg sample was subjected to ultrasonic extraction in 1 mL of ultrapure water containing 2 μg/mL of internal standards for 60 min at 4°C to ensure maximal metabolite recovery. Following centrifugation (12,000 rpm, 10 min, 4°C), the supernatant was diluted 10‐fold prior to analysis. Chromatographic separation was performed on a Waters ACQUITY UPLC I‐Class system (Waters, Milford, MA, USA) using an HSS T3 column (100 × 2.1 mm, 1.8 μm) at 45°C. The mobile phase consisted of 0.1% formic acid in water (A) and acetonitrile (B), with a linear gradient from 5% to 100% B over 14 min at 0.35 mL/min. Mass spectra were acquired on a Thermo Q‐Exactive HF‐X mass spectrometer in both positive and negative ion modes (full‐scan/dd‐MS^2^, *m*/*z* 100–1500). Resolution was set to 60,000 (MS1) and 15,000 (MS2), with stepped normalized collision energies of 10, 20, and 40 eV. Compound identification was performed by matching retention times, accurate masses, and MS/MS spectra against the LuMet‐TCM database (Shanghai OE Biotech Co. Ltd).

### 2.3 Identification of Active Components and Target Prediction

Identified constituents were cross‐referenced with the HERB (http://herb.ac.cn/) and TCMSP (https://www.tcmsp‐e.com/) databases. Drug‐likeness was evaluated using SwissADME (https://swissadme.ch/), with compounds selected that met ≥ 3 of 5 drug‐likeness rules and exhibited high predicted gastrointestinal absorption. Molecular targets were predicted using SwissTargetPrediction (top 20 per compound) and standardized via UniProt biodata platform (https://www.uniprot.org).

### 2.4 Collection of AS‐Associated Genes

AS‐related genes were retrieved from GeneCards (https://www.genecards.org/) using the keyword “atherosclerosis.” Targets with a relevance score ≥ 1 were included. Overlapping genes between GXBD‐predicted targets and disease‐associated genes were identified as potential therapeutic targets.

### 2.5 Network Construction and Functional Enrichment

Compound‐target and protein–protein interaction (PPI) networks were constructed using Cytoscape (version 3.9.1). PPI data were obtained from the STRING database (https://cn.string‐db.org/) (confidence score > 0.7). Gene Ontology (GO), Kyoto Encyclopedia of Genes and Genomes (KEGG) enrichment analyses were performed on intersecting targets using the DAVID database (Sherman et al. 2022). The enrichment results provided functional insights into the biological processes and signaling pathways that GXBD may modulate.

### 2.6 Transcriptomic Data Integration

Differentially expressed genes (DEGs) associated with AS were obtained from three Gene Expression Omnibus (GEO) datasets (GSE100927, GSE43292, GSE28829) using a linear regression model adjusted for available covariates. Genes with an adjusted *p* < 0.01 were considered significant. These DEGs were then intersected with GXBD‐predicted targets and PPI network nodes, and key hub genes were prioritized using the cytoHubba plugin in Cytoscape (MCC algorithm) to identify critical regulators potentially mediating the effects of GXBD.

### 2.7 Animal Experiments

The entire animal handling protocol adhered strictly to the NIH Guide for the Care and Use of Laboratory Animals. Furthermore, all experimental protocols received stringent approval from the Ethics Committee of Henan University of Chinese Medicine (Approval No. DWLL202202009). Sample size (*n* = 8 per group) was determined based on a power analysis (G*Power v3.1), aiming for > 80%, power at *α* = 0.05, utilizing effect sizes derived from previous studies on lipid‐lowering interventions in ApoE^−/−^ mice (Xie et al. 2025; Zhou et al. 2025). Forty‐eight male ApoE^−/−^ mice (8 weeks old, 20–30 g) were housed under specific pathogen‐free (SPF) conditions, were purchased from SPF (Beijing) biotechnology Co. Ltd. After one week of acclimatization, mice were randomly allocated into six groups: Control (Normal Diet), Model (High‐Fat Diet, HFD), three GXBD dose groups (Low: 0.625 g/kg; Medium: 1.25 g/kg; High: 2.50 g/kg), and Simvastatin (20 mg/kg). The HFD (21% fat, 0.15% cholesterol) and treatments were administered daily by oral gavage for 14 weeks. GXBD dosages were calculated based on clinical equivalent doses using body surface area normalization.

The complete six‐group design was utilized to establish the dose–response relationship and confirm efficacy. For the subsequent mechanistic studies (microbiota, metabolomics, and barrier function), we employed a focused design analyzing the Control, Model, and High‐dose GXBD (2.50 g/kg) groups. This strategy allowed for greater sequencing depth and resource allocation to characterize the specific mechanism of the optimal GXBD dose. Simvastatin was excluded from the omics analysis to avoid confounding variables, as its single‐target mechanism (HMG‐CoA reductase inhibition) is distinct from the multi‐component, microbiota‐modulating action of the herbal decoction.

### 2.8 Histological Assessment of Aortic Lesions

Upon reaching the experimental endpoint, the mice were first anesthetized via an intraperitoneal injection of pentobarbital sodium (40 mg/kg). Subsequently, perfusion was performed using PBS, after which the aortas were meticulously excised and preserved by fixing in a 4% paraformaldehyde solution. To facilitate morphological assessment, the aortic roots were subjected to serial sectioning. These sections were then stained with hematoxylin and eosin (H&E) for general plaque architecture, Oil Red O to quantify lipid deposition, and Masson's trichrome for the evaluation of collagen content.

### 2.9 Transmission Electron Microscopy (TEM)

Aortic samples (1 × 1 × 2 mm) were fixed in 2.5% glutaraldehyde, post‐fixed in osmium tetroxide, dehydrated in graded ethanol, embedded in epoxy resin, and sectioned for uranyl acetate–lead citrate staining. Ultrastructural changes in endothelial and smooth muscle cells were examined with a JEOL transmission electron microscope, providing insights into plaque stability.

### 2.10 Biochemical Analyses

Serum levels of TG, TC, LDL‐C, and HDL‐C were determined using off‐the‐shelf commercial assay kits. The concentrations of inflammatory cytokines, which include TNF‐α (E‐EL‐M3063), IL‐6 (E‐EL‐M0044), and IL‐1β (E‐EL‐M0037), were accurately quantified. This was performed using ELISA kits procured from Elabscience (Wuhan, China). Hepatic and fecal cholesterol levels were also measured.

### 2.11 Gut Microbiota Analysis

The V3‐V4 hypervariable region of the bacterial 16S rRNA gene was successfully amplified from extracted fecal DNA. Subsequent sequencing of these amplicons was executed utilizing the Illumina HiSeq 6000 platform. Bioinformatic analysis was performed using QIIME2 (version 2020.2). Alpha and beta diversity metrics were calculated. Linear Discriminant Analysis Effect Size (LEfSe) (v 1.1.2) was employed to identify differential taxa. Functional prediction was conducted using PICRUSt2 to generate COG and KEGG pathway classifications.

### 2.12 Targeted Bile Acid Metabolomics

Bile acids in fecal samples were quantified using LC–MS/MS (SCIEX QTRAP 6500+). Briefly, 20 mg of feces was homogenized in methanol containing internal standards, then precipitated at −20°C, centrifuged, and filtered. Chromatography was performed on a Waters HSS T3 column using a gradient of acetonitrile and aqueous ammonium acetate with 0.01% acetic acid. Multiple reaction monitoring (MRM) was used for BA quantification, employing a 16‐point calibration curve (0.1–4000 ng/mL, *R* ^2^ > 0.99).

### 2.13 Microbiota–Metabolite Correlation Analysis

Redundancy analysis (RDA) was conducted using CANOCO 5.0 to examine the relationships between gut microbiota and key bile acid metabolites (VIP > 1.0 from OPLS‐DA). Spearman's rank correlation was used to assess the associations between differential microbial genera (with relative abundance ≥ 0.1%) and significantly altered bile acids. Correlation networks were visualized using the “pheatmap (v1.0.12)”, “ggplot2 (v3.4.4)” and “igraph (v1.5.1)” packages in R (v4.2.0) and mapped in Cytoscape (version 3.9.1).

### 2.14 Quantitative Reverse Transcription PCR (RT‐qPCR)

Total RNA isolation was performed on snap‐frozen aortic tissues. The extraction process utilized the TRIzol reagent obtained from Thermo Fisher Scientific, USA. Subsequently, the purity and concentration of the extracted RNA were quantified using spectrophotometry. For cDNA synthesis, a 1 μg aliquot of the total RNA was reverse‐transcribed using the PrimeScript RT Master Mix kit (Takara Bio, Shiga, Japan). Quantitative PCR was performed on a CFX96 Real‐Time PCR Detection System (Bio‐Rad, Hercules, CA, USA) using SYBR Premix Ex Taq II (Takara Bio, Shiga, Japan). Gene expression levels were normalized to GAPDH and calculated using the 2^−ΔΔ*Ct*^ method. Primers for ZO‐1, Occludin, MMP9, CASP3, PPARG, SIRT1, and MTOR were designed based on published sequences (Table 2) and synthesized by Sangon Biotech (Shanghai, China).

**TABLE 2:** Primer sequences.

Gene	Sequence (5′ to 3′)
ZO‐1	
Forward	TGG ACG TGG AAC CAG AAG AGG
Reverse	TGA GAA GAG GAG ACA GCT TAG GC
Occludin	
Forward	GAG GAA CTG CTG GCA AAA GGA TGG
Reverse	GAC GCT TAT GTT GTT GCT GAT GGC
IL‐6	
Forward	GAG AGG AGA CTT CAC AGA GGA TAC C
Reverse	TCA TTT CCA CGA TTT CCC AGA GAA C
IL‐1β	
Forward	TCG CAG CAG CAC ATC AAC AAG
Reverse	TCC ACG GGA AAG ACA CAG GTA G
TGF‐β	
Forward	ACC GCA ACA ACG CCA TCT ATG AG
Reverse	GGC ACT GCT TCC CGA ATG TCT G
β‐Actin	
Forward	AGC CAT GTA CGT AGC CAT CC
Reverse	CTC TCA GCT GTG GTG GTG AA

### 2.15 Statistical Analysis

Data analysis was performed using SPSS 21.0 (IBM Corp., Armonk, NY, USA) and GraphPad Prism 9.0. Normality of data distribution was verified using the Shapiro–Wilk test. Data conforming to a normal distribution are presented as mean ± SD. Specifically, body weight, serum lipids (TC, TG, LDL‐C, HDL‐C), and inflammatory cytokines were analyzed using one‐way ANOVA. Data not following a normal distribution (such as relative abundance of microbiota and pathology scores) are presented as median with interquartile range and were analyzed using the non‐parametric Kruskal‐Wallis test. Spearman correlation was applied for microbiota–metabolite associations. A *p*‐value < 0.05 was considered statistically significant.

## 3 Results

### 3.1 Chemical Profiling and Network‐Based Target Prediction of GXBD

**FIGURE 1:**
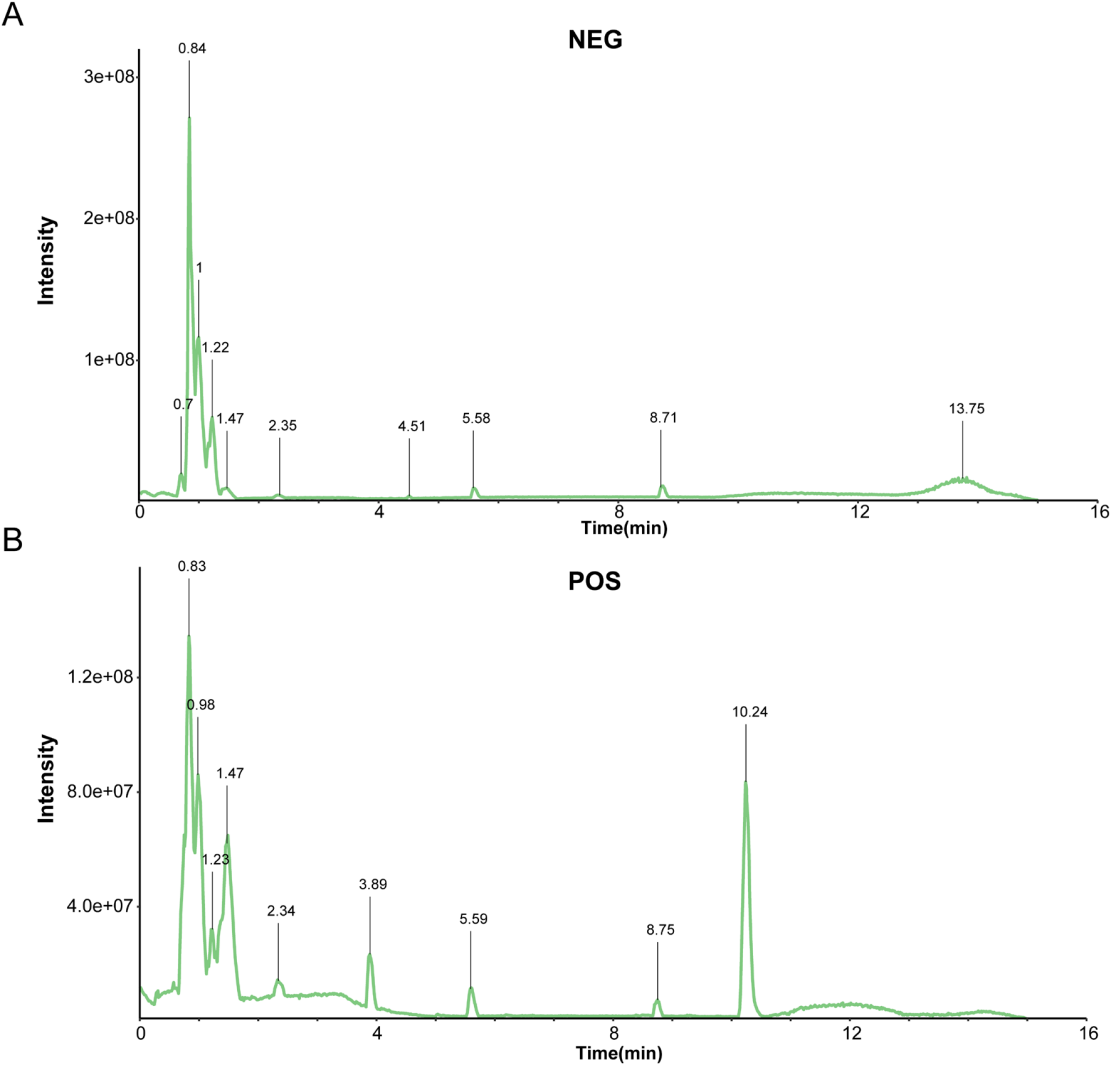
Chemical profiling of Gualou Xiebai Banxia Decoction (GXBD) using UHPLC‐QE‐MS/MS. (A) Total ion chromatogram obtained in negative ion mode. (B) Total ion chromatogram obtained in positive ion mode.

**FIGURE 2:**
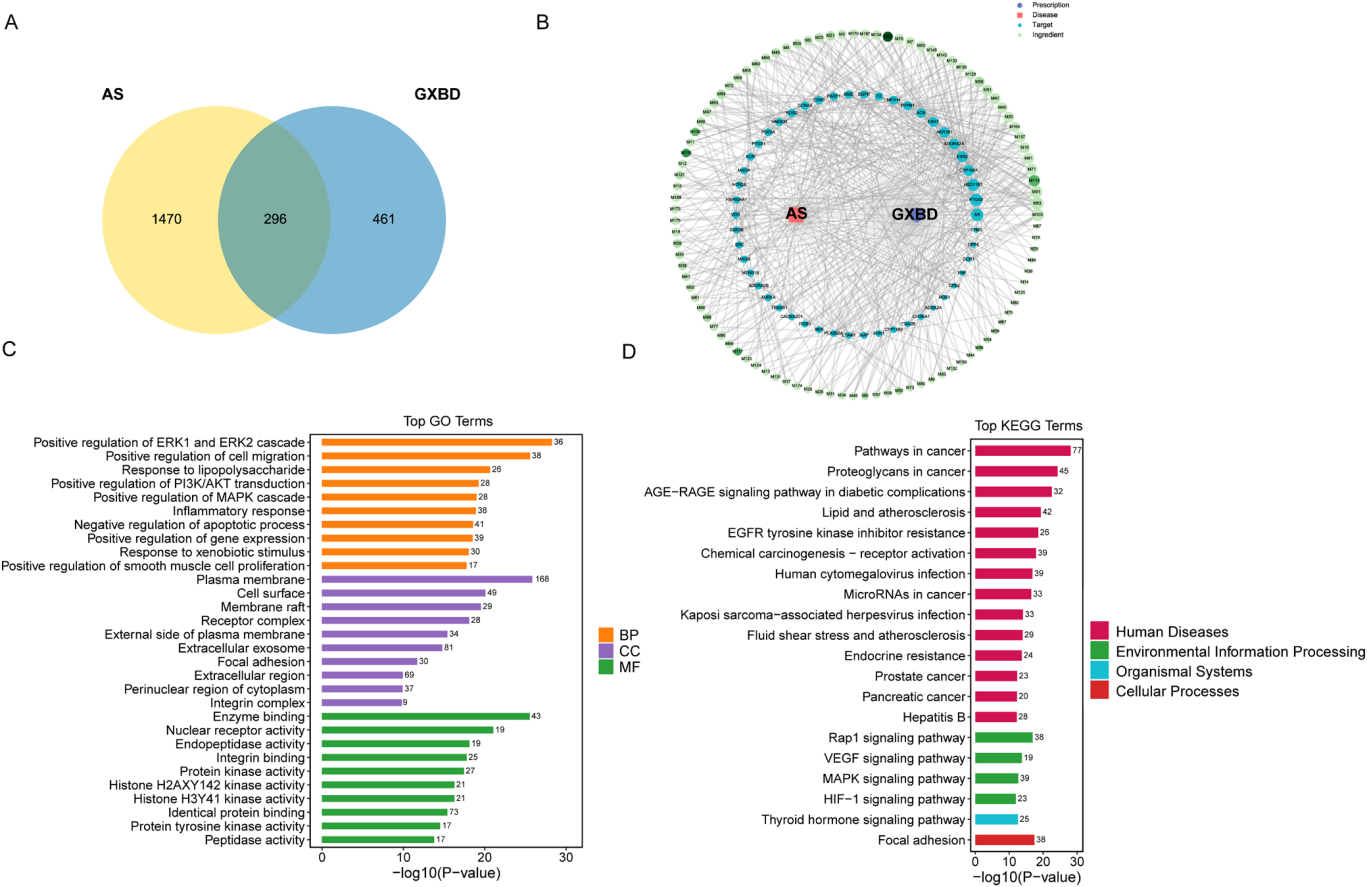
Network pharmacology‐based prediction of Gualou Xiebai Banxia Decoction (GXBD) targets associated with AS. (A) Venn diagram showing the overlap between GXBD‐predicted targets and AS‐related genes. (B) Integrated compound–target–disease network. (C) Gene Ontology (GO) enrichment analysis of the intersecting targets. (D) Kyoto Encyclopedia of Genes and Genomes (KEGG) pathway enrichment analysis.

A total of 348 constituents were identified in the chemical profile of GXBD using UHPLC‐QE‐MS/MS analysis. The identified profile was dominated by bioactive classes including flavonoids (e.g., luteolin), steroidal saponins (e.g., timosaponin BII), and cucurbitacins (e.g., cucurbitacin D) (Figure 1). This multi‐component chemical profile aligns with the complex pharmacological actions expected of a classical Chinese herbal formula (Chen, Wang, et al. 2023; Xiang et al. 2022). From this total pool, 34 compounds met the SwissADME criteria for drug‐likeness and gastrointestinal absorption. Subsequent network pharmacology analysis mapped these active ingredients to 296 unique AS‐related targets (Figure 2A,B). Functional enrichment analysis (KEGG) indicated that these targets were significantly enriched in lipid metabolism and inflammatory pathways, particularly the PI3K‐Akt, MAPK, and PPAR signaling cascades (Figure 2C,D).

### 3.2 Transcriptomic Integration and Experimental Validation of Predicted Targets

**FIGURE 3:**
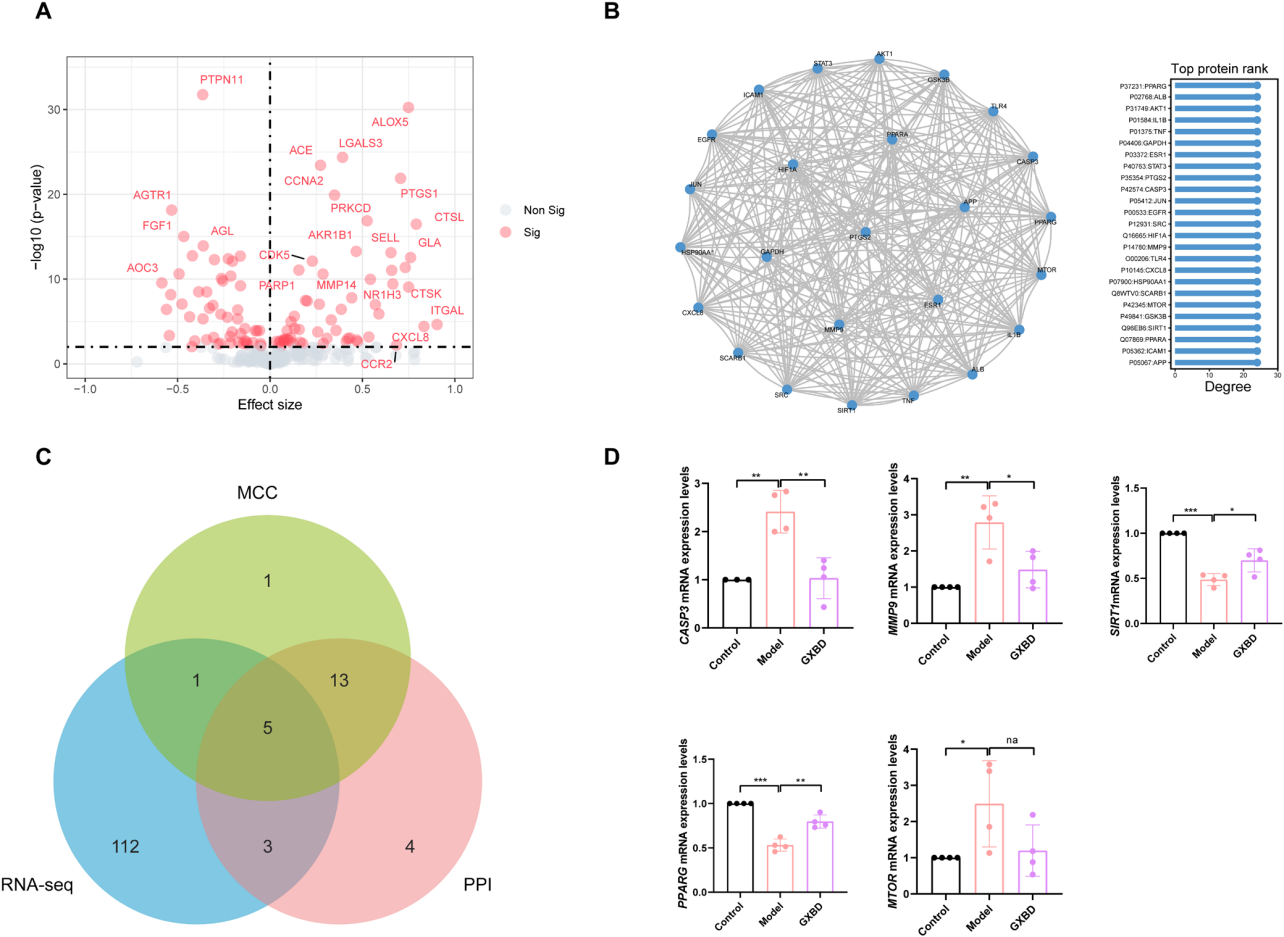
Identification and validation of core therapeutic targets of Gualou Xiebai Banxia Decoction (GXBD) in AS. (A) Volcano plot showing the differential expression of GXBD‐predicted targets based on three integrated human AS transcriptomic datasets. (B) Protein–protein interaction (PPI) network of intersecting targets constructed using the STRING database. (C) Venn diagram illustrating the overlap among hub targets identified by network pharmacology, transcriptomic analysis, and PPI topology. (D) RT‐qPCR analysis of selected hub genes (*MMP9*, *CASP3*, *MTOR*, *PPARG*, *SIRT1*) in aortic tissues. Data are presented as mean ± SD (*p < 0.05, **p < 0.01, ***p < 0.001).

Integration of three human AS transcriptomic datasets (GSE100927, GSE43292, and GSE28829) with GXBD‐predicted targets and PPI network nodes yielded five cross‐validated core targets: *MMP9*, *CASP3*, *MTOR*, *PPARG*, and *SIRT1* (Figure 3A–C). Experimental validation via RT‐qPCR in aortic tissues demonstrated that GXBD intervention, particularly at medium and high dosages, significantly downregulated the mRNA expression of *CASP3* and *MMP9* compared to the model group (*p* < 0.05). Conversely, the expression levels of *PPARG* and *SIRT1* were significantly upregulated (*p* < 0.05, Figure 3D). No statistically significant change was observed in *MTOR* expression following treatment.

### 3.3 GXBD Improves Lipid Metabolism and Promotes Plaque Stabilization

**FIGURE 4:**
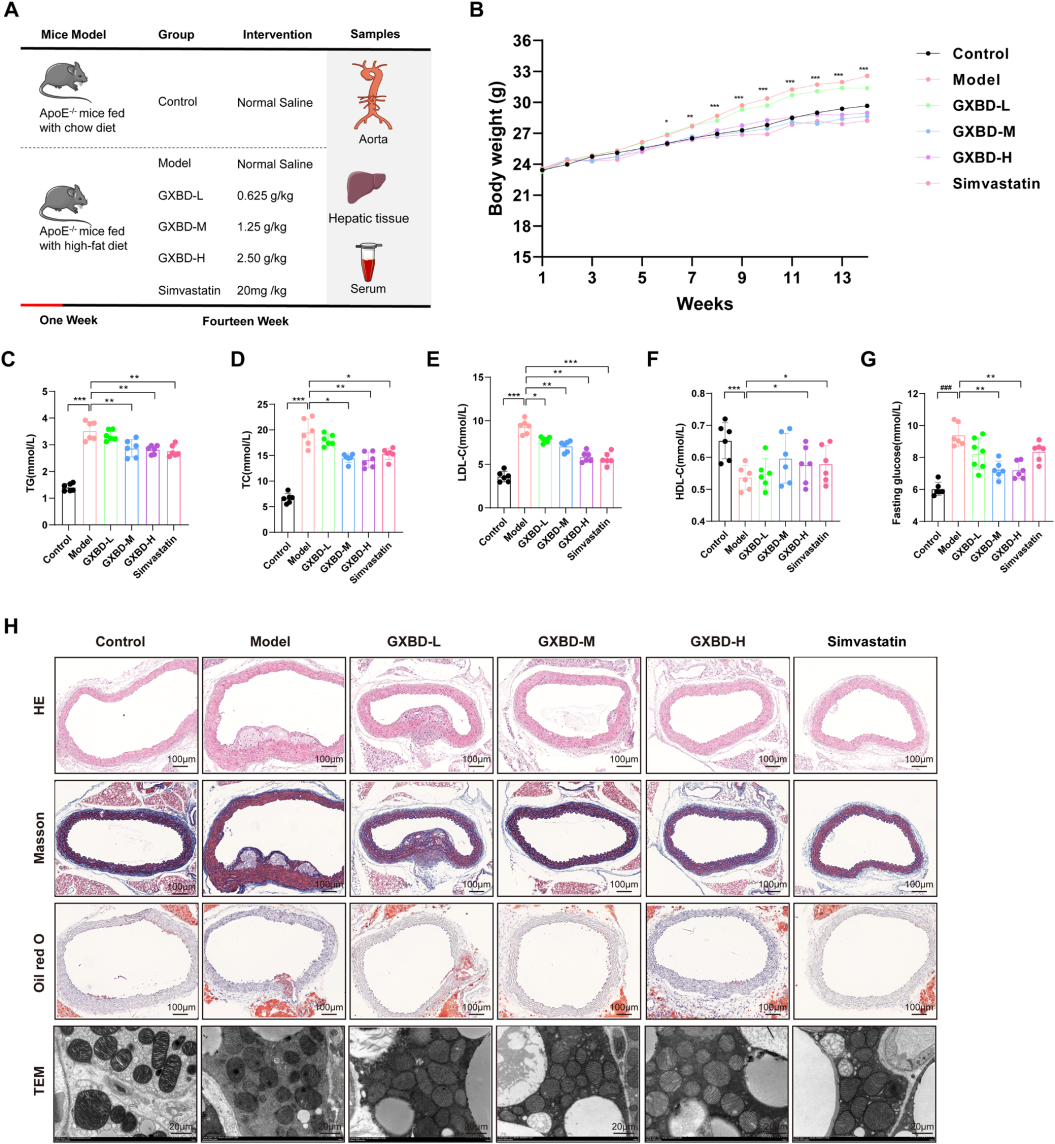
Effects of Gualou Xiebai Banxia Decoction (GXBD) on lipid metabolism, glucose homeostasis, and atherosclerotic plaque characteristics in *ApoE* ^*−/−*^ mice. (A) Schematic of the experimental design, intervention strategy, and sampling timeline. (B) Longitudinal monitoring of body weight changes over the 14 weeks. (C–F) Serum lipid profile analysis, including (C) Triglycerides (TG), (D) Total cholesterol (TC), (E) LDL‐cholesterol (LDL‐C), and (F) HDL‐cholesterol (HDL‐C). (G) Fasting blood glucose levels across experimental groups. (H) Representative histological images of aortic roots stained with Hematoxylin and Eosin (H&E) for plaque morphology, Oil Red O for lipid deposition, and Masson's trichrome for collagen content. Transmission electron microscopy (TEM) images show mitochondrial ultrastructure in endothelial cells. Data are presented as mean ± SD (*p < 0.05, **p < 0.01, ***p < 0.001).

To investigate the protective effects of GXBD against dyslipidaemia and atherosclerosis, the study examined the impact of 14 weeks of high‐fat diet (HFD) feeding with or without GXBD intervention (Figure 4A). Compared to the model group, GXBD administration significantly attenuated HFD‐induced weight gain, particularly at medium and high doses (*p* < 0.05) (Figure 4B). Systemic lipid analysis revealed that GXBD treatment significantly reduced serum TC, TG, and LDL‐C levels while increasing HDL‐C (*p* < 0.05), achieving efficacy comparable to that of Simvastatin (Figure 4C–F). Additionally, GXBD treatment effectively regulated glucose homeostasis, as evidenced by a significant reduction in fasting blood glucose levels compared to the model group (Figure 4G). Histopathological examination of aortic roots demonstrated that HFD feeding induced extensive lipid‐rich plaques. Treatment with GXBD‐M and GXBD‐H significantly decreased both total plaque area and lipid deposition (*p* < 0.05). While Masson's trichrome staining showed a non‐significant reduction in total collagen content, TEM revealed that GXBD preserved endothelial ultrastructure, characterized by reduced mitochondrial swelling and cristae disruption (Figure 4H). Based on these comprehensive phenotypic outcomes, the High‐dose group (GXBD‐H) was prioritized for the subsequent multi‐omics mechanistic investigation.

### 3.4 GXBD Alleviates Hepatic Steatosis, Improves Intestinal Barrier Integrity, and Reduces Inflammation

**FIGURE 5:**
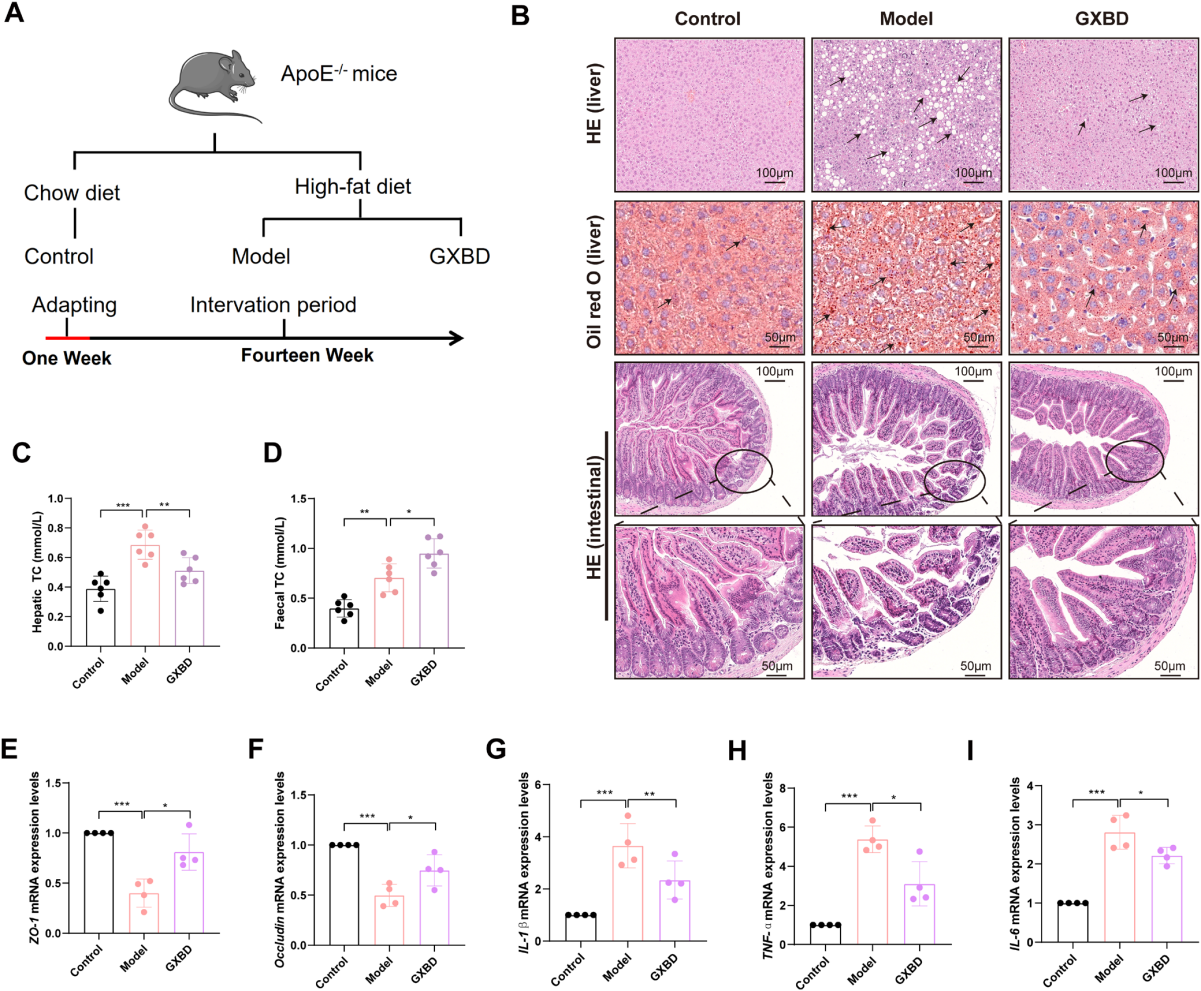
Effects of Gualou Xiebai Banxia Decoction (GXBD) on hepatic steatosis, intestinal barrier integrity, and inflammation in *ApoE* ^*−/−*^ mice. (A) Schematic diagram of the experimental grouping and intervention timeline for the mechanism‐focused study. (B) Representative histological images of liver sections stained with H&E and Oil Red O, and ileal sections stained with H&E. (C) Quantification of hepatic total cholesterol (TC) levels. (D) Quantification of fecal total cholesterol (TC) levels. (E–I) Relative mRNA expression levels of ileal tight junction proteins (*ZO‐1* and *Occludin*) and pro‐inflammatory cytokines (*IL‐1β*, *IL‐6*, and *TNF‐α*). Data are presented as mean ± SD (*p < 0.05, **p < 0.01, ***p < 0.001).

Further analysis was conducted to examine the effects of GXBD on the intestinal barrier, flora and metabolites of mice with AS that was induced by a high‐fat diet (Figure 5A). Histopathological assessment using H&E and Oil Red O staining revealed pronounced hepatocellular steatosis in the model group. GXBD treatment significantly alleviated this pathology, restoring hepatic architecture and reducing lipid deposition (Figure 5B). In the ileum, the model group exhibited villus blunting and epithelial barrier disruption, whereas GXBD treatment preserved mucosal architecture and reduced inflammatory cell infiltration (Figure 5B). Biochemical analysis confirmed a significant reduction in hepatic TC levels (*p* < 0.05) (Figure 5C) and a concomitant increase in fecal TC excretion (*p* < 0.05) compared to the model group (Figure 5D). Furthermore, GXBD induced a significant upregulation in the mRNA expression of tight junction proteins *ZO‐1* and *Occludin* (*p* < 0.05) (Figure 5E,F) and significantly downregulated the pro‐inflammatory cytokines *IL‐1β*, *IL‐6*, and *TNF‐α* in ileal tissues (*p* < 0.05) (Figure 5G–I).

### 3.5 GXBD Reshapes Gut Microbiota Composition and Functional Potential

**FIGURE 6:**
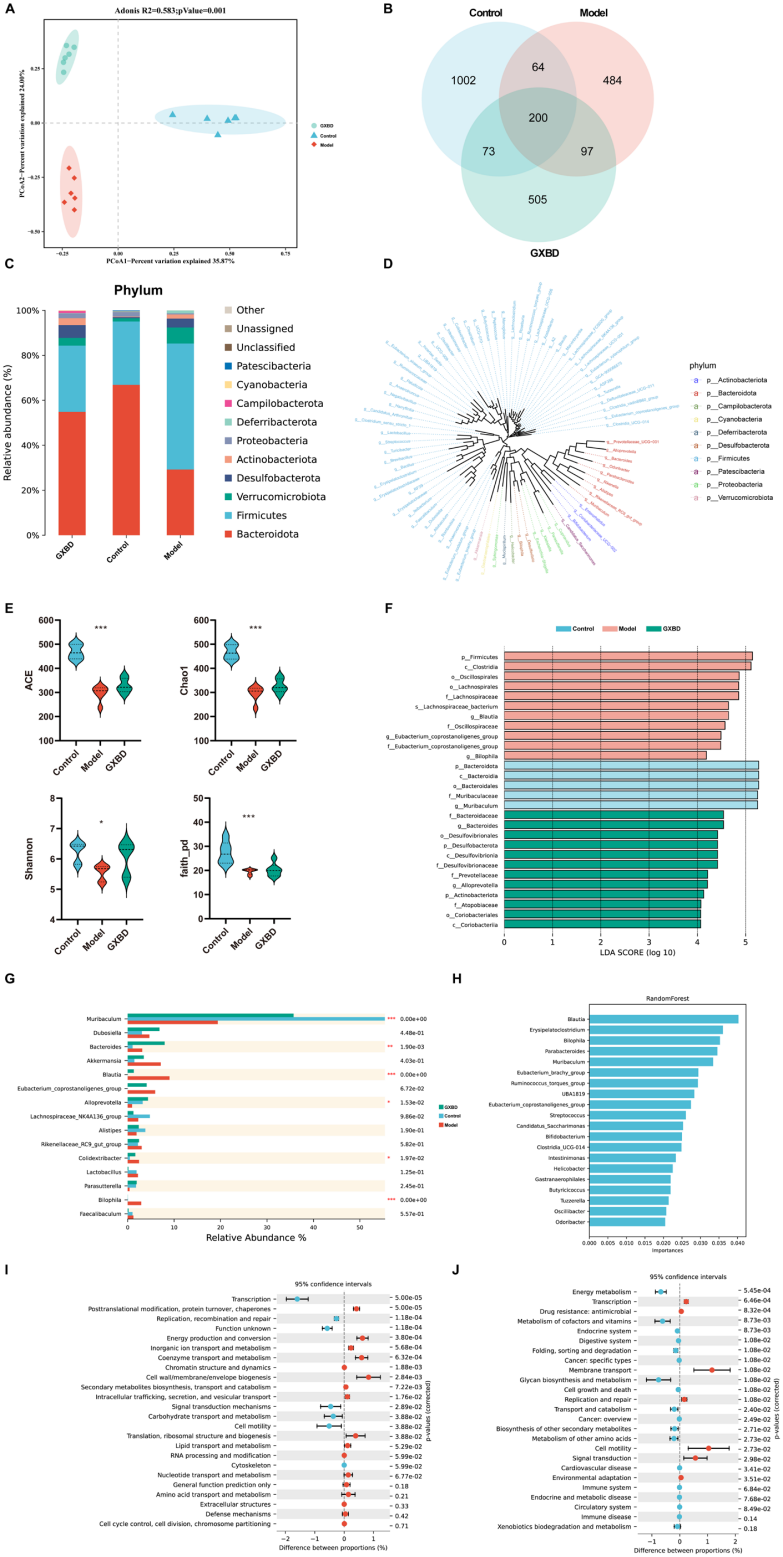
Effects of Gualou Xiebai Banxia Decoction (GXBD) on gut microbiota composition and predicted functional pathways in ApoE^−/−^ mice. (A) Principal Coordinates Analysis (PCoA) based on the Jaccard distance matrix. (B) Venn diagram showing shared and unique amplicon sequence variants (ASVs). (C) Relative taxonomic composition at the phylum level. (D) Phylogenetic tree of gut microbial communities at the genus level. (E) Alpha diversity indices, including ACE, Chao1, Shannon, and Faith's PD. (F) Linear discriminant analysis Effect Size (LEfSe) analysis identifying differentially enriched taxa (LDA score > 4). (G) Relative abundance of predominant genera across groups. (H) Random forest analysis ranking the top discriminatory taxa. (I) Functional prediction of microbial communities using PICRUSt2 (COG categories). (J) Predicted KEGG pathways (Level 2) showing functional differences across groups. For panels (E) and (G), data are presented as violin plots, with internal lines indicating the median and quartiles. Statistical significance was determined using the Kruskal‐Wallis test (*p < 0.05, **p < 0.01, ***p < 0.001).

Principal coordinates analysis (PCoA) demonstrated a distinct structural separation between the GXBD and Model groups, indicating substantial remodeling of the gut microbiota (Figure 6A). Venn analysis showed that all three groups shared 200 core amplicon sequence variants (ASVs) (Figure 6B). At the phylum level, *Bacteroidota*, *Firmicutes*, and *Verrucomicrobiota* dominated the microbial community (Figure 6C,D). Compared with the control group, model mice exhibited significantly reduced microbial richness and diversity indices (ACE, Chao1, Simpson, Faith's PD) (*p* < 0.05). GXBD treatment significantly restored these diversity metrics (*p* < 0.05) (Figure 6E).

Linear discriminant analysis (LEfSe, LDA > 4) revealed that the model group was enriched with *Blautia* and *Bilophila*, whereas GXBD treated mice exhibited significantly increased abundance of *Bacteroides* and *Alloprevotella* (Figure 6F). At the genus level, the marked elevation of *Colidextribacter* in the model group was significantly reversed by GXBD (*p* < 0.05), while *Muribaculum* abundance increased (Figure 6G). Random forest analysis identified *Blautia*, *Erysipelatoclostridium*, and *Bilophila* as the top discriminative taxa (Figure 6H). Predictive metagenomic profiling (PICRUSt2) indicated that GXBD altered COG pathways related to transcriptional regulation and protein turnover (Figure 6I). KEGG pathway analysis predicted enhanced energy metabolism, cofactor/vitamin metabolism, and endocrine system–associated functions in the GXBD group (Figure 6J).

### 3.6 GXBD Modulates Fecal Bile Acid Metabolism

**FIGURE 7:**
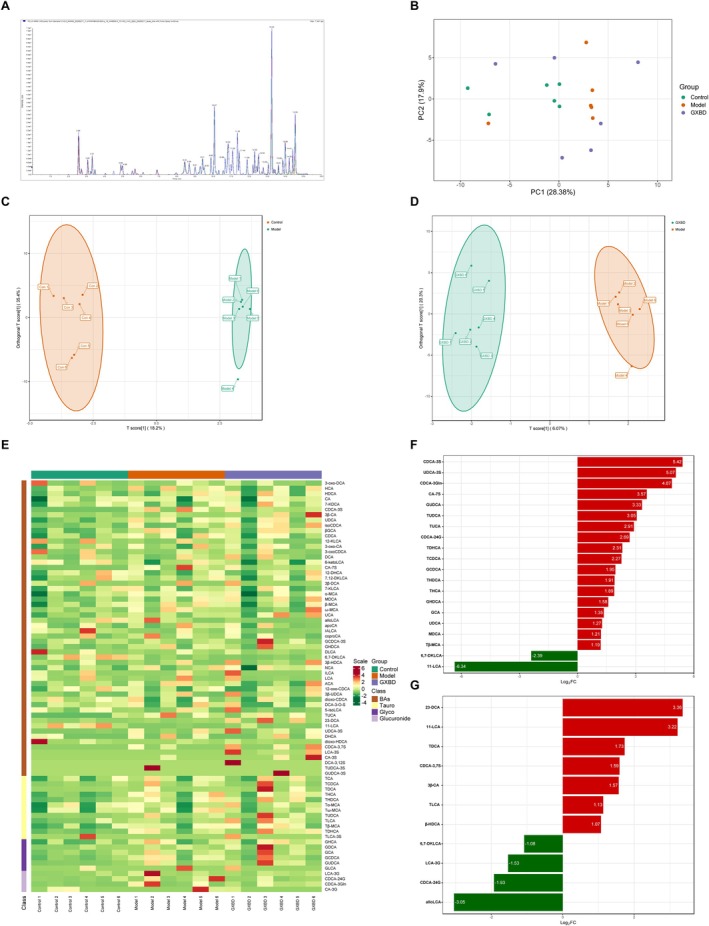
Effects of Gualou Xiebai Banxia Decoction (GXBD) on fecal bile acid (BA) composition in high‐fat diet–fed *ApoE* ^*−/−*^ mice. (A) Total ion chromatograms (TIC) of fecal bile acids acquired by LC–MS/MS. (B) Principal Component Analysis (PCA) score plot based on fecal BA profiles. (C, D) Orthogonal Partial Least Squares Discriminant Analysis (OPLS‐DA) score plots comparing (C) Model vs. Control groups and (D) GXBD versus Model groups. (E) Hierarchical clustering heatmap showing the relative abundance of differentially regulated bile acids. (F) Relative abundance of differential bile acid species across Control, Model, and GXBD groups (VIP > 1.0, Fold Change > 2.0). (G) Relative abundance of differential bile acid species identified between GXBD and model groups. Data are presented as mean ± SD (*p < 0.05, **p < 0.01, ***p < 0.001).

Total ion chromatograms acquired by LC–MS/MS reflected the overall fecal BA composition (Figure 7A). Principal component analysis (PCA) demonstrated clear separation among the control, model, and GXBD groups, with the GXBD group exhibiting a distinct shift toward a profile resembling that of Controls (Figure 7B). Orthogonal Partial Least Squares Discriminant Analysis (OPLS‐DA) further substantiated these metabolic distinctions, showing that GXBD‐treated mice clustered closer to the control group than to the untreated model mice (Figure 7C,D). Hierarchical clustering heatmaps visually confirmed that GXBD partially reversed HFD‐induced alterations in the BA spectrum (Figure 7E).

Key discriminant BAs were identified based on Variable Importance in Projection (VIP > 1) and fold‐change filtering (FC ≥ 2). Compared with the control group, model mice exhibited significantly increased levels of CDCA‐3S, UDCA‐3S, and CDCA‐3Gln, alongside decreased levels of 11‐LCA and 6,7‐DKLCA (Figure 7F). Notably, GXBD administration significantly increased the abundance of 23‐DCA, 11‐LCA, and TDCA, while significantly reducing 6,7‐DKLCA, LCA‐3G, and CDCA‐24G compared to the model group (*p* < 0.05) (Figure 7G).

### 3.7 Correlation Analysis of the Microbiota—Bile Acid Axis

**FIGURE 8:**
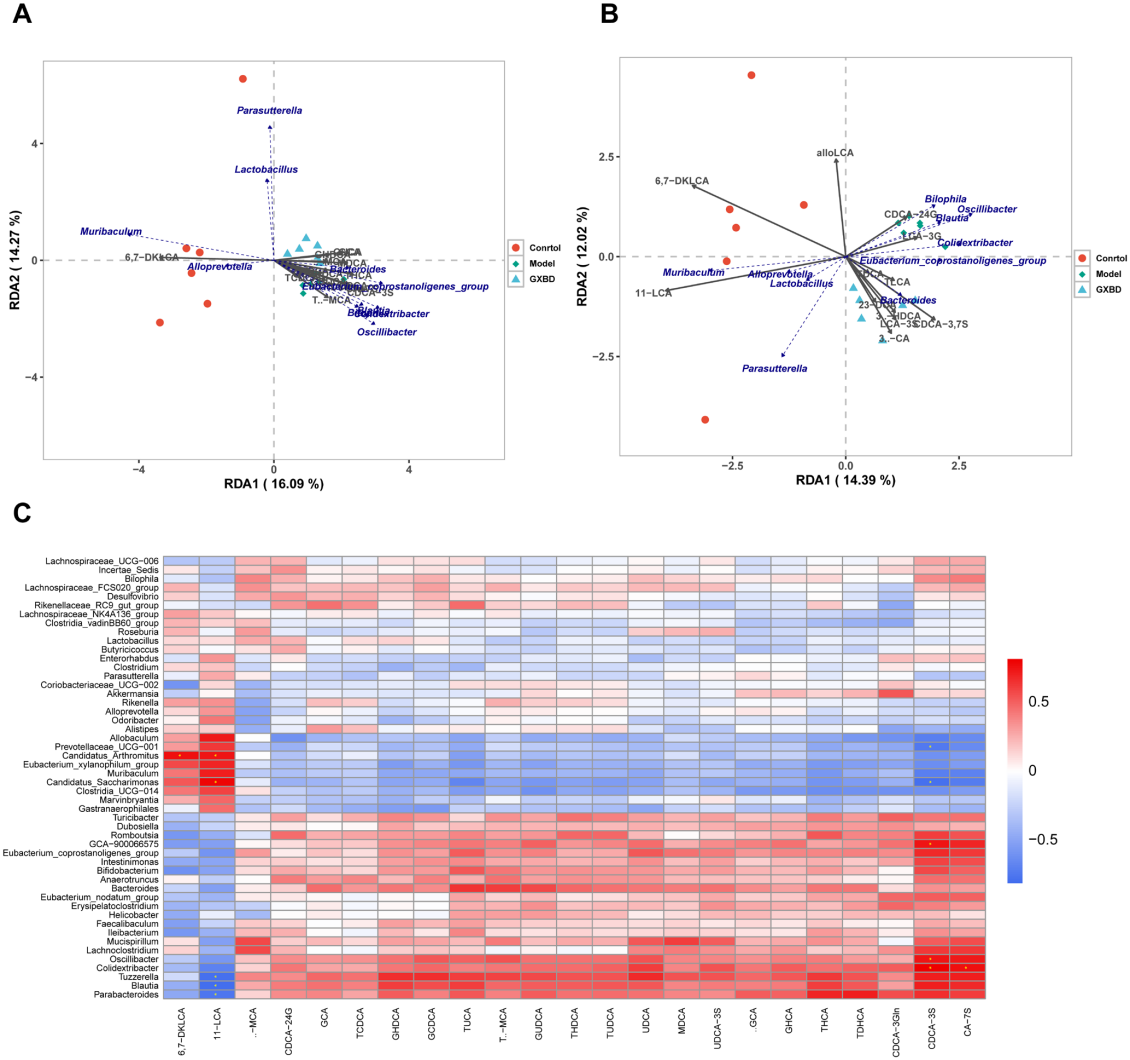
Associations between gut microbiota and fecal bile acid (BA) profiles in *ApoE* ^*−/−*^ mice treated with Gualou Xiebai Banxia Decoction (GXBD). (A) Redundancy analysis (RDA) triplot showing the relationship between gut microbiota composition and fecal BA profiles in the Control and Model groups. (B) RDA triplot showing the relationship between gut microbiota composition and fecal BA profiles in the GXBD and Model groups. (C) Heatmap of Spearman's correlation coefficients between differentially abundant gut microbial genera and fecal BA species (*p < 0.05).

Redundancy analysis (RDA) revealed distinct associations between fecal bile acid profiles and specific bacterial taxa across experimental groups (Figure 8A,B). Spearman correlation analysis further identified key interactions between discriminant taxa and bile acids. *Colidextribacter* exhibited strong positive correlations with CDCA‐3S (*r* = 0.806) and CA‐7S (*r* = 0.752). *Blautia* showed a negative correlation with 11‐LCA (*r* = −0.820) but a positive correlation with CDCA‐3S (*r* = 0.705). *Bacteroides* demonstrated a positive correlation with TUCA (*r* = 0.629) and a negative correlation with 11‐LCA (*r* = −0.528). *Alloprevotella* was positively correlated with 11‐LCA (*r* = 0.322) and negatively correlated with T‐MCA (*r* = −0.511) (*p* < 0.05) (Figure 8C).

## 4 Discussion

While lipid‐lowering therapies have significantly evolved, AS continues to dominate global morbidity rates, leaving many patients exposed to residual cardiovascular risk driven by unresolved metabolic inflammation. To confront this clinical gap, we investigated the mechanistic role of the classical Chinese formula, GXBD using an integrated multi‐omics approach in *ApoE* ^−/−^ mice. Our findings indicate that GXBD confers robust anti‐atherosclerotic protection, manifested by improved systemic metabolism and enhanced plaque stability. Mechanistically, we identified that these therapeutic benefits hinge on the remodeling of the “gut microbiota–secondary bile acid–host” metabolic axis. This study bridges the gap between the TCM concept of “Phlegm Turbidity” in *Xiong Bi* (Chest Obstruction) and modern microbiome‐targeted therapeutic strategies (Riaz et al. 2023).

The therapeutic potency of GXBD stems from its complex phytochemical composition. Using UHPLC‐QE‐MS/MS profiling, we identified key bioactive constituents, including luteolin, loliolide, timosaponin BII, and cucurbitacin D (Chen, Wang, et al. 2023; Xiang et al. 2022), which collectively contribute to its pleiotropic effects. Through an integration of network pharmacology and transcriptomics, we identified *MMP9*, *CASP3*, *PPARG*, and *SIRT1* as pivotal therapeutic targets. Subsequent validation revealed that GXBD promotes a two‐pronged mechanism: it upregulates the metabolic checkpoints *PPARG* and *SIRT1* (Montaigne et al. 2021; Sazdova et al. 2024; Zhou et al. 2024), suggesting a restoration of systemic homeostasis beyond mere lipid lowering, while concurrently dampening the pro‐inflammatory and apoptotic drivers *MMP9* and *CASP3* (Saito et al. 2022). This molecular profile directly corroborates our histological observations of enhanced plaque stability. Ultimately, this synergistic modulation underscores the holistic nature of GXBD, which addresses atherosclerosis by combining metabolic correction with vascular protection.

Moving from molecular signaling to tissue architecture, our histological assessments offer physical proof of plaque stabilization. While the model group exhibited profound collagen deposition, the matrix structure was aberrant and disorganized—a hallmark of pathological remodeling rather than structural integrity. Crucially, our data suggest that GXBD confers stability by preventing the proteolytic erosion of the fibrous cap, rather than by simply stimulating collagen synthesis. This preservation strategy, driven by *MMP9* suppression, effectively lowers rupture risk despite the absence of significant gains in total collagen mass (Björkegren and Lusis 2022; Fusco et al. 2025). Concurrently, TEM revealed that GXBD mitigates mitochondrial swelling and cristae disruption in endothelial cells, a morphological benefit that mechanistically correlates with the suppression of *CASP3*. By arresting stress‐induced endothelial apoptosis, GXBD preserves vascular barrier competence, culminating in a clinically stable, quiescent plaque phenotype.

A notable mechanistic finding of this study is the remodeling of the gut microbiota–bile acid axis (Yoshida et al. 2018). GXBD administration induced a profound shift in gut microbial composition, characterized by the enrichment of beneficial genera such as *Bacteroides* and *Alloprevotella* (Huang et al. 2021; Wang et al. 2022), and the reduction of pro‐inflammatory taxa, including *Blautia* and *Bilophila* (Chen, Mou, et al. 2023; Fang et al. 2022). Crucially, this microbial restructuring was tightly correlated with a favorable alteration of the BA pool: specifically, the significant elevation of protective secondary bile acids (11‐LCA and 23‐DCA) and the reduction of cytotoxic CDCA derivatives (Cheng et al. 2024; Yang et al. 2022). This association is biologically plausible, as secondary BAs are exclusive products of microbial transformation. While CDCA has been implicated as a significant risk factor for AS, LCA derivatives are increasingly recognized as protective ligands that regulate host metabolism. Therefore, our data highlight the often‐underappreciated role of specific microbial‐derived secondary BA species in mediating the anti‐AS effects of TCM.

Integrating these metabolomic and transcriptomic findings allows us to propose a coherent signaling cascade (Cortés and Eckel 2022; Qi et al. 2022). Protective secondary BAs, such as those elevated by GXBD (11‐LCA and 23‐DCA), are established ligands for nuclear receptors like FXR and TGR5 (Fiorucci et al. 2020). The observed upregulation of *PPARG* and *SIRT1* in aortic tissues provides a plausible downstream link, as BA signaling via TGR5 is known to crosstalk with PPAR pathways to resolve chronic inflammation and enhance lipid efflux. Based on our multi‐omics correlations and existing literature, we hypothesize the following mechanistic pathway: the enrichment of GXBD in *Bacteroides* facilitates the production of secondary BAs; these metabolites likely act as signaling molecules to activate host metabolic receptors, coinciding with the upregulation of *PPARG*/*SIRT1* and the suppression of *MMP9*/*CASP3*. This functional integration positions GXBD as a potential “bile acid‐modulating agent” that leverages the gut‐liver‐vascular axis for plaque stabilization.

## 5 Limitations

The main strength of this study is the use of an integrated multi‐omics approach. This method successfully links the traditional TCM concept of “Phlegm Turbidity” with the modern “gut microbiota–bile acid–host” metabolic axis. By focusing on the GXBD‐H, we obtained a detailed profile of the therapeutic mechanism. However, there are some limitations. First, although we observed strong associations between microbial changes and host improvements, future studies utilizing fecal microbiota transplantation (FMT) in germ‐free mice are warranted to validate the causal nature of this axis. Second, our molecular analysis focused on specific targets (e.g., *MMP9*, *SIRT1*); future studies using comprehensive proteomics would provide a broader view of the signaling pathways. Finally, including a simvastatin group in future multi‐omics analyses would help to distinguish the specific effects of the herbal formula from the general effects of lipid‐lowering drugs.

## 6 Conclusion

Collectively, this study leverages multi‐omics profiling to map the anti‐atherosclerotic landscape of GXBD. Our data construct a hierarchical model where plaque stabilization—phenotypically anchored by the reciprocal regulation of *MMP9/CASP3* and *PPARG/SIRT1*—is inextricably linked to systemic metabolic correction. A defining feature of our findings is the identification of a gut‐liver signaling loop, where GXBD re‐engineers the microbial ecosystem to prioritize the synthesis of protective secondary bile acids, specifically 11‐LCA and 23‐DCA. This “gut microbiota–secondary bile acid–host” axis not only provides a modern scientific rationale for the TCM theory of treating “Phlegm Turbidity” but also positions GXBD as a viable microbiome‐modulating candidate for targeting residual cardiovascular risk.

## Author Contributions

**Rutao Bian:** conceptualization, methodology, investigation, formal analysis, data curation, writing – original draft. **Li Zhang:** validation, resources, visualization, investigation, writing – review and editing. **Jun Zhu:** conceptualization, supervision, project administration, funding acquisition, writing – review and editing. **Xuegong Xu:** conceptualization, supervision, project administration, funding acquisition, writing – review and editing.

## Funding

This work was supported by the Henan Provincial Science and Technology Tackling Project (262102311236) and the Key Project of the Henan Academy of Traditional Chinese Medicine (2025ZKY013).

## Ethics Statement

Ethical oversight for all animal experiments was provided by the Ethics Committee of Laboratory Animal Science at Henan University of Chinese Medicine. The entire protocol received formal approval under the designation No. DWLL202202009.

## Conflicts of Interest

The authors declare no conflicts of interest.

## Data Availability

The complete datasets that were produced and subsequently analyzed in the context of this study can be obtained directly from the corresponding author when a justifiable and reasonable request is submitted.
